# Publisher Correction: Ballistic geometric resistance resonances in a single surface of a topological insulator

**DOI:** 10.1038/s41467-018-02964-z

**Published:** 2018-02-05

**Authors:** Hubert Maier, Johannes Ziegler, Ralf Fischer, Dmitriy Kozlov, Ze Don Kvon, Nikolay Mikhailov, Sergey A. Dvoretsky, Dieter Weiss

**Affiliations:** 1 Institute of Experimental and Applied Physics, University of Regensburg, 93053 Regensburg, Germany; 2 A.V. Rzhanov Institute of Semiconductor Physics, 630090 Novosibirsk, Russia; 3 Novosibirsk State University, 630090 Novosibirsk, Russia

Correction to: *Nature Communications* 10.1038/s41467-017-01684-0, published online 08 December 2017

In the original version of this Article, the second and third sentences of the first paragraph of the “Gate voltage and antidot period dependencies” section of the Results originally incorrectly read “The characteristic evolution of the sheet resistance *ρ*_□_=*ρ*_□_ (*B*=0), with *V*_g_ shown for three antidot samples and an unpatterned reference sample in Fig. 3a. The maxima of *ρ*_xx_, located between *V*_g_~0.5 and 1 V, reflect the charge neutrality point (CNP), corresponding to an *E*_F_ position located slightly in the valence band (see band structure in Fig. 3b).” In the corrected version, “${\mathrm{\rho }}_\square = {\mathrm{\rho }}_\square (B = 0)$” is replaced by “${\mathrm{\rho }}_\square = {\mathrm{\rho }}_{{\mathrm{xx}}}(B = 0)$”, and “The maxima of ${\mathrm{\rho }}_{{\mathrm{xx}}}$” is replaced by “The maxima of ${\mathrm{\rho }}_\square$”.

These errors have been corrected in both the PDF and HTML versions of the Article.

